# Validation of a Chinese version of disease specific quality of life scale (HFS-36) for hemifacial spasm in Taiwan

**DOI:** 10.1186/1477-7525-7-104

**Published:** 2009-12-24

**Authors:** Yen-Chu Huang, Jun-Yu Fan, Long-Sun Ro, Rong-Kuo Lyu, Hong-Shiu Chang, Sien-Tsong Chen, Wen-Chuin Hsu, Chiung-Mei Chen, Yih-Ru Wu

**Affiliations:** 1 Department of Neurology, Chang Gung Memorial Hospital, Chiayi, Taiwan; 2 Department of Neurology, Chang Gung Memorial Hospital, Linkou, Taiwan; 3 Chang-Gung University College of Medicine, Taipei, Taiwan; 4 Department of Nursing, Chang Gung Institute of Technology, Tao-Yuan, Taiwan

## Abstract

**Background and object:**

There was no Chinese questionnaire to evaluate the health-related quality of life (HRQoL) in patients with hemifacial spasm (HFS). In this study, we aimed to validate a new disease-specific HRQoL scale for HFS (HFS-36) in Chinese version, and compared it to SF-36, a generic HRQoL scale.

**Patients and Methods:**

The HFS-36 Chinese version was modified from English version of HFS-30, including subscales of mobility, activities of daily living (ADL), emotional well-being, stigma, social support, cognition, bodily discomfort, and communication. All the items were scored on the 5-point scales, ranging from 0(never) to 4(always). Patients with HFS were asked to answer HFS-36 and SF-36 questionnaires on the same day before and 6-8 weeks after Botulinum toxin (BTX) injections, respectively. The reliability and validity of HFS-36 scale were evaluated statistically.

**Results:**

Totally, 103 patients (68 females; 35 males) were recruited in this study, with a mean age of 57.6 ± 11.5 years and a mean duration of HFS for 7.6 ± 5.8 years. The intra-class correlation (ICC) and Cronbach's α were over 0.7 in the majority of items. HFS-36 showed a good correlation to HFS severity before BTX treatment and a significant improvement of subscale scoring after BTX treatment. HFS-36 also had a significant correlation to the mental health of SF-36.

**Conclusions:**

The Chinese version of HFS-36 demonstrated a good reliability and validity in subscales of motility, ADL, emotion well-being, stigma and bodily discomfort. The HRQoL was significantly improved after BTX treatment assessed by HFS-36 or SF-36. Compared to SF-36, HFS-36 scale was more sensitive and specific to evaluate the HRQoL in HFS.

## Introduction

Hemifacial spasm (HFS) is characterized by involuntary contractions of the facial muscles innervated by the ipsilateral facial nerve, usually without any identifiable etiology. It has been recognized as a result of compression of the facial nerve at the root exit zone by an anatomical or pathological structure. Though not life threatening, patients with HFS may complain of social embarrassment and somatic discomforts, including interference with vision, eye irritation, tearing, difficulty in reading and driving, dysarthria, facial paresthesia, hearing of "clicking" or a "ticking" sound, trismus, etc. Most patients feel the movement persisted during sleep. Such problems invariably reduce patients' quality of life (QoL).

Application of Botulinum toxin (BTX) is currently regarded as a preferred treatment [1,2]. The treatment outcomes include relief of facial contractions and satisfaction with various aspects of their life quality. Health-related quality of life (HRQoL) is an important outcome criterion of medical interventions [3], but was barely studied in patients with HFS due to lack of appropriate instrument. Reimer *et al *had used SF-36 and National Eye Inventory Visual Function Questionnaire (NEI-VFQ) to evaluate the global and disease-specific HRQoL respectively in patients with blepharospasm and HFS, and they found the HRQoL in these patients were significantly impaired compared with healthy controls [4]. However, NEI-VFQ scale was not designed specifically for HFS, and the generic scale (SF-36) may not fully represent the impact on their QoL. Tan *et al *had designed a disease-specific HRQoL scale (HFS-30) to evaluate the response of BTX treatment [5], which showed a good correlation of severity of HFS in some subscales. However, some questions were not relevant and several important components, such as sleep quality and bodily complaints, were not included. Later, they developed a short self-rating scale (HFS-7) which showed a correlation to SF36 [6]. Hauser *et al *added an important item related to sleep quality (HFS-8) to evaluate the QoL after microvascular decompression for HFS [7]. Currently, there was no Chinese questionnaire to evaluate the HRQoL in patients with HFS. In this study, we aimed to validate a new disease-specific QoL scale for HFS in Chinese version, and compared it to SF-36.

## Materials and methods

This study was approved by the institutional review board of Chang Gung Memorial Hospital and it enrolled patients fulfilled the criteria of: (1) a clinical diagnosis of primary HFS, (2) under Botulinum toxin type A treatment (Botox^® ^(Allergan, USA)), and (3) could understand and answer questions properly. Patients who had concomitant disability, severe medical problems (such as malignancy, organ failure, severe lung diseases, etc.) and other neurological diseases (like blepharospasm, Parkinson's disease, etc.), were all excluded. They were treated and evaluated by an experienced neurologist (Wu YR) in the neurology clinics. The potential complications of BTX treatment had been informed and they consented to participate in this study. All the patients received BTX injection, ranging from 15 - 40 unites.

In the beginning, there were 32 patients in the test-retest reliability exam. They answered HFS-36 at fourth and sixth week after BTX treatment. After analyzing test-retest reliability, 103 patients, including initial 32 patients, were recruited in this study. They were asked to answer SF-36 and HFS-36 questionnaires on the same day before and 6-8 weeks after BTX treatment, respectively. The severity of HFS was assessed at the same time.

### SF-36 Questionnaire

The SF-36 is a multipurpose and widely used short-form health survey with 36 questions, which includes eight domains: physical functioning(PF), role limitations due to physical health (RP), role limitations due to emotional problems (RE), vitality(VT), mental health(MH), social functioning(SF), bodily pain(BP), and general health(GH) [8]. Among them, PF, RP, BP and GH belong to physical health, whereas RE, VT, MH and SF belong to mental health. The SF-36 Taiwan standard version has been validated in our population [9].

### HFS-36 Chinese Version Questionnaire

The HFS-36 Chinese version was modified from English version of HFS-30. The designed process includes two steps. The first step was the linguistic validation of a HFS-30 Chinese version including forward and backward translation. This process was conducted to make sure the conceptually equivalent to the English version, as well as clear and easy to understand. The HFS-30 English version was translated separately into Chinese by two native Chinese speakers with good knowledge of English, which were translated back into English by another Chinese professional translator who had excellent knowledge of Chinese and English. The back-translation was compared with the original English version by a native English speaker. We repeated back-translations and made further modifications until a consensus was reached.

The second step was to examine whether the HSF-36 Chinese version has an appropriate items to reflect the construct (QoL of HFS patients). As the authors mentioned in introduction section, sleep quality and bodily complaints are not included in HFS-30 English version. We added a new domain including 5 items for bodily discomfort, and an item in the stigma domain to HFS-30. The "draft" HFS-36 Chinese version was finalized. Three neurologists rated each scale item in terms of its relevance to the underlying construct on a 4-point ordinal scale. Both item-level and scale-level content validity index (CVI) were computed and all values were at least of 0.8. Item 2,4,8,9 were rewording or add other options due to culture difference. For example, item 4 "riding motorcycle or bicycle" was added since majority of our patients rode motorcycle or bicycle instead of driving. All the changes were underlined in the table 1. The finalized HFS-36 Chinese version contained 8 subscales, including mobility (items 1-5), activities of daily living (ADL) (items 6-10), emotional well-being (items 11-17), stigma (items 18-22), social support (items 23-25), cognition (items 26-28), bodily discomfort (items 29-33), and communication (items 34-36). All items were scored on a five point scale ranging from 0(never) to 4 (always). The answers to these questions represented how patients feel in recent 2-3 weeks.

**Table 1:** The items of HFS-36 and its reliability (test--retest)

Subscales/items of HFS-36	ICC
**Mobility**	
1. Had difficulty doing leisure activities	0.82
2. Had difficulty looking after your home, **such as fixing or cleaning your house**	0.82
3. Had difficulty at work	0.84
4. Had difficulty driving/**riding motorcycle/riding bicycle**	0.83
5. Had difficulty crossing the road	0.50
**Activities of Daily Living**	
6. Had difficulty reading	0.89
7. Had difficulty watching television/movie	0.79
8. Had difficulty using computer/**or dialing phone**	0.70
9. Had difficulty writing/**or using chopsticks**	0.55
10. Had difficulty doing household chores	0.83
**Emotional Well-being**	
11. Felt depressed	0.86
12. Felt weepy and tearful	0.90
13. Felt angry or bitter	0.83
14. Felt anxious of going blind	0.69
15. Felt fearful of treatment	0.90
16. Felt worried of getting a stroke	0.73
17. Felt worried of losing your job	0.68
**Stigma**	
**18. Concern about your appearance**	0.92
19. Avoided eye contact	0.86
20. Avoided eating and drinking in public	0.72
21. Felt embarrassed about having the condition	0.92
22. Felt worried about other's reactions to you	0.79
**Social support**	
23. Had problems with close relationship	0.66
24. Did not have support from spouse or partner	0.35
25. Did not have support from family and friends	0.39
**Cognition**	
26. Had problems with concentration	0.59
27. Had problems with headaches	0.72
28. Had problems with giddiness	0.66
**Bodily discomfort**	
**29. Had problems with tinnitus or hearing impairment**	0.74
**30. Felt difficulty to fall asleep or had poor sleep quality**	0.76
**31. had sensation of facial numbness or pain**	0.76
**32. had problem of eye irritation, tearing or photophobia**	0.84
**33. had problem of drooling or swallowing difficulty**	0.85
**Communication**	
34. Had difficulty with speech	0.82
35. Felt unable to communicate properly	0.77
36. Felt ignored by people	0.85

Emboldened words: the difference from original HFS-30

ICC: intraclass correlation coefficient

### Assessment of severity of HFS and response to treatment

The severity of HFS was scored based on the five point scale (0: normal, 1: slight disability, 2: moderate disability, without functional impairment, 3: moderate disability, with functional impairment, and 4: severely incapacitated). The severity was assessed by Dr. Wu YR in the neurology clinics, before and 6~8 weeks post BTX treatment. Because HFS tended to vary in different situations, they were evaluated in a period time when answering questionnaires or under their interview. The response of BTX treatment was represented as: (1) the difference of spasm severity or (2) percentage improvement of spasm severity. Because patient's self-rating or perception regarding treatment response was strongly related to and confounded the scoring of HRQoL, this part was not included in judging the effectiveness after BTX injection, which was different from what was used by Tan [5].

### Statistical analysis

The Statistical Program for Social Sciences (SPSS) statistical software (version 16.0) (SPSS Inc., Chicago) was used for data analysis and the significant level was set up at *p *< 0.05. An intra-class correlation (ICC) approach was used to examine the test-retest reliability of HFS-36. ICC in single measure, two-way mixed model, was applied since the instrument would only be administered once to a subject at one period of time [10]. The ICC greater 0.7 indicated good reliability. For each subscales, the score was standardized and re-scaled from 0 to 100 [Subscale score: (Sum of the item scoring in the subscale)*25/Item numbers in the subscale]. Reliability testing was used to evaluate the internal consistency of each subscale and Cronbach's α over 0.7 represented good reliability. Independent sample *t *test was used to evaluate the difference between subscale scores of male and female. Spearman's rank correlation was applied to determine the correlation between HFS severity and HFS-36 scoring, treatment response and HFS-36 scoring difference, HFS severity and SF-36 scoring, as well as treatment response and difference of SF-36 scoring. Paired sample *t*-test was applied to determine the difference before and after treatment in SF-36 and HFS-36. The items of HFS-36 were ranked according to the mean difference before and after treatment.

## Results

Totally, 103 patients (68 females; 35 males) were recruited in this study, with a mean age 57.6 ± 11.5 years (ranging 30-86 years). The mean duration of HFS was 7.6 ± 5.8 years (ranging 0.6-39.5 years), with right-side predominant (55 patients). The mean severities of HFS were 2.83 ± 0.9 (ranging 1-4) before and 0.67 ± 0.6 (ranging 0-3) after treatment. The proportion of each severity for HFS before treatment were 26.2%(severity 4), 38.8%(severity 3), 27.2%(severity 2), 7.8%(severity 1), 0%(severity 0), whereas those after treatment were 0%(severity 4), 1.0%(severity 3), 6.8%(severity 2), 50.5%(severity 1), 41.7%(severity 0). There was 37 patients (35.9%) reported minor side effects related to BTX treatment, including drooling (12.6%), blurred vision (7.8), tearing (5.8%), eyelid weakness(4.9%), facial weakness(2.9%) and ptosis(2.9%). These side effects all disappeared later.

ICC of each item in the test-retest reliability was listed in table 1; among them, there were 9 items not greater than 0.7, including: item 5 in motility; item 9 in ADL; item 14 and 17 in emotional well-being; items 23-25 in social support; items 26 and 28 in cognition. The mean of each subscale score and their Cronbach's α were listed in table 2. The Cronbach's α was lowest in the subscale of social support (0.67). Subscales of social support and communication had lower scoring before treatment (1.1 and 2.8 respectively in table 2). Females rated significant higher scores than males in subscale of emotional well-being, stigma and cognition (table 2). This study used Spearman's rank correlation to evaluate the correlation of HFS severity and subscale scores of HFS-36 before treatment, and it revealed statistically positive correlations in the subscales of motility, ADL, emotional well being, and bodily discomfort (Table 3). Most of subscale scores of HFS-36 improved significantly after treatment, except subscales of social support (Table 3). However, the improvement (response of BTX treatment) did not significantly correlate to the change of HFS-36 scores in each subscale.

**Table 2:** Reliability of scale (internal consistency) and mean of the subscale scores before BTX treatment

			Mean of the subscale scores		
			
Subscales	Item number	Cronbach's α	Total	Male	Female
**Mobility**	1-5	0.91	13.5	11.2	14.7
**Activities of daily living**	6-10	0.92	13.1	9.4	15.0
**Emotional well-being**	11-17	0.91	15.7	6.6	20.3*
**Stigma**	18-22	0.91	31.7	23.5	35.8*
**Social support**	23-25	0.67	1.1	1.2	1.1
**Cognition**	26-28	0.85	13.9	9.6	16.1*
**Bodily discomfort**	29-33	0.75	16.9	13.9	18.4
**Communication**	34-36	0.81	2.8	1.7	3.3

* indicates a significant difference compared to male, *p *< 0.05

**Table 3:** Correlation of HFS-36 subscale and severity of HFS before BTX treatment and difference of HFS-36 before and after treatment

		Correlation of HFS-36 subscale and severity of HFS		Difference of HFS-36 before and after treatment	
		
Subscales	Item number	Spearsman's Correlation	*p*-value	Mean difference #	*p*-value
**Mobility**	1-5	0.23*	0.023	12.1*	<0.0001
**Activities of daily living**	6-10	0.25*	0.011	10.5*	<0.0001
**Emotional well-being**	11-17	0.24*	0.013	8.8*	<0.0001
**Stigma**	18-22	0.06	0.566	20.4*	<0.0001
**Social support**	23-25	0.15	0.144	0.6	0.07
**Cognition**	26-28	0.13	0.208	10.5*	<0.0001
**Bodily discomfort**	29-33	0.25*	0.010	8.3*	<0.0001
**Communication**	34-36	0.19	0.058	2.6*	0.007

* indicates a significant difference, *p *< 0.05

# the mean difference of each subscales scoring before and after treatment

In SF-36, the scores were improved after BTX treatment in domains of PF (p = 0.04), RP (p < 0.001), RE (p < 0.001), VT (p < 0.001), MH (p < 0.001). The Spearman's rank correlations of HFS-36 and SF-36 before and after Botox treatment were listed in table 4. Except subscales of social support and communication, other subscales of HFS-36 had good correlation to mental health of SF-36 no matter before or after Botox treatment (table 4).

**Table 4:** Correlation of HFS-36 and SF-36 before/after BTX treatment

Subscales	PF	RP	RE	VT	MH	SF	BP	GH
**Mobility**	-0.20/-0.25*	-0.36*/0.11	-0.36*/-0.13	-0.23*/-0.39*	-0.16/-0.33*	-0.22*/-0.23*	-0.17/-0.17	-0.10/-0.09
**ADL**	-0.25*/-0.13	-0.26*/-0.17	-0.28*/-0.42*	-0.36*/-0.39*	-0.25*/-0.31*	-0.21*/-0.32*	-0.24*/-0.20	-0.19/-0.26*
**Emotional well-being**	-0.10/-0.17	-0.11/-0.17	-0.19/-0.07	-0.31*/-0.29*	-0.42*/-0.41*	-0.33*/-0.28*	-0.12/-0.26*	-0.25*/-0.26*
**Stigma**	-.013/0.04	-0.08/-0.07	-0.13/-0.27*	-0.26*/-0.18	-0.38*/-0.36*	-0.41*/-0.30*	-0.03/-0.15	-0.19/-0.15
**Social support**	0.06/-0.15	-0.26*/0.07	-0.01/-0.28*	-0.05/-0.09	-0.09/-0.13	-0.11/**-0.21**	0.03/-0.14	-0.04/-0.09
**Cognition**	-0.12/-0.12	-0.19/-0.10	-0.25*/-0.24*	-0.29/*-0.26*	-0.36*/-0.13	-0.32*/-0.23*	-0.14/-0.10	-0.23*/-0.12
**Bodily discomfort**	-0.32*/-0.31*	-0.22*/-0.14	-0.23*/-0.29*	-0.34*/-0.22*	-0.22*/-0.18	-0.12/-0.28*	-0.24*/-0.26*	-0.32*/-0.26*
**Communication**	-0.17/-0.13	-0.03/-0.04	-0.15/-0.08	0.02/0.01	0.05/-0.14	-0.11/-0.03	0.08/-0.11	<0.01/-0.01

PF: physical functioning; RP: role limitations due to physical health; RE: role limitations due to emotional problems; VT: vitality; MH: mental health; SF: social functioning; BP: bodily pain; GH: general health

* indicates a significant difference, *p *< 0.05

Paired sample *t*-test was applied to compare HFS-36 scores before and after BTX treatment, and only six items were not statistically significant. The items were ranked according to the mean difference of each item score, and a greater mean difference indicated more sensitive to reflect the changes after treatment (table 5).

**Table 5:** Ranking of each item by the mean difference before and after treatment

Ranking	Items of HFS-36	Mean difference	p value	ICC	% reaching floor	% reaching ceiling	HFS 7	HFS 10
1	19. Avoided eye contact	0.99	<0.001	0.86	8.7	40.8	*	#

2	11. Felt depressed	0.92	<0.001	0.86	4.9	40.8	*	#

3	18. Concern about your appearance	0.89	<0.001	0.92	13.6	28.2		#

4	21. Embarrassed about the condition	0.85	<0.001	0.92	6.8	52.4	*	#

5	26. Problems with concentration	0.76	<0.001	0.59	0	47.6		

6	1. Difficulty doing leisure activities	0.74	<0.001	0.82	1.0	57.3		#

7	22. Worried about other's reactions	0.70	<0.001	0.79	5.8	62.1	*	

8	20. Avoided eating in public	0.65	<0.001	0.72	4.9	56.3		

9	3. Had difficulty at work	0.61	<0.001	0.84	0	63.1		#

10	13. Felt angry or bitter	0.59	<0.001	0.83	1.9	67.0		#

11	6. Had difficulty reading	0.57	<0.001	0.89	0	54.4	*	#

12	7. Difficulty watching television	0.54	<0.001	0.79	0	54.4	*	

13	30. Difficulty to fall asleep	0.50	<0.001	0.76	1.0	59.2		#

14	4. Difficulty driving/riding bicycle	0.48	<0.001	0.83	0	66.0	*	

15	9. Difficulty writing	0.44	<0.001	0.55	1.0	70.9		

16	29. Problems with tinnitus	0.41	<0.001	0.74	0	48.5		

17	32. Problem of eye irritation	0.41	0.001	0.84	1.9	46.6		#

18	2. Difficulty looking after your home	0.36	<0.001	0.82	0	73.8		

% reaching floor: % reaching a score of 4 before treatment (the worst functional status.)

% reaching ceiling: % reaching a score of 0 before treatment (the best functional status)

* HFS-7 items.

# Items suggested in the short scale for hemifacial spasm, HFS-10.

## Discussion

The outcome of BTX treatment includes the relieving of hemifacial spasm and the improvement of HRQoL. HFS-36, derived from English version of HFS-30, is the first Chinese version scale in assessing HRQoL in patients with HFS. Several items in the subscales of motility (item 2 & 4) and ADL (item 8 & 9) of HFS-30 were modified to fit the lifestyle in Taiwan. A new subscale of bodily discomfort contained 5 items were added to create the HFS-36.

The reliability of HFS-36 was examined by the ICC of test-retest exams and items with lower ICC value (<0.7) were largely observed in subscales of social support and cognition (Table 1). These items with less favorable ICC may also be related to the fluctuation of HFS symptoms from day to day especially under stress and anxiety despite the test-retest was performed in the duration with stationary effect of BTX. Nevertheless, most of the items in HFS-36 were reliable and reproducible. Except subscale of social support, the Cronbach's α in the other subscales were all over 0.7 indicating good internal consistency (Table 2). The top three of the mean subscale score before treatment were stigma (31.7), bodily discomfort (16.9), and emotional well-being (15.7) (Table 2), representing greater impact on HRQoL, whereas subscales of social support and communication had lower score indicating less influence. Moreover, females rated higher scores than males, with significant difference in subscales of emotional well-being, stigma and cognition. It may hint that HFS annoyed females more than males.

Among the subscale in HFS-36, there was a significant correlation of HFS severity and subscale scores, including motility, ADL, emotional well-being and bodily discomfort (Table 3); subscales scores of stigma and cognition did not correlate significantly despite their mean scores were higher before treatment(scored 31.7 and 13.9 respectively in table 2). However, scores of these two subscales were significantly improved after BTX treatment (Table 3). Therefore, the spasm severity was not in accord with the impairment of HRQoL. For example, patients with mild symptoms of spasm severity may still have enormous embarrassment (items 18-22) or feel difficult in concentration (item 26).

Unlike the results reported by Tan [5], the improvement of HFS-36 scores was not proportional to the changes of severity scales in our study. The discrepancy may be due to the different measure of the treatment response. Tan *et al *adopted patient's self-perception as part of the response of treatment, whereas we only used the changes of spasm severity as treatment response. Since the self-perception of treatment response strongly influenced the self-rating of HRQoL, and thus will confound the results of correlation. Indeed, our measure also had some potential bias. The symptoms of HFS are intermittent and may vary with different emotional state. There may be some discrepancies between the spasm severity scoring and the exact disease severity whatever the time we evaluate. In addition, the HFS severity scale is not a validated scale, so a cautious interpretation is advised.

SF-36 was the most wildly used generic scale to evaluate HRQoL. The scores were significantly improved after BTX in domains of PF, RP, RE, VT, and MH. This result proved that BTX treatment could improve HRQoL mainly in the mental health. When comparing HFS-36 to SF-36 both before and after Botox treatment, subscales of motility, ADL, emotional well-being, stigma and cognition were significantly correlated to the SF-36, especially in domains of mental health (RE, VT, MH & SF). On the other hand, subscale of bodily discomfort was significantly correlated to both mental and physical health (Table 4). However, subscales of social support and communication rarely correlated to SF-36 and the two subscales did not have significant correlation to severity of HFS before BTX, either. Therefore, subscales of social support and communication in HFS-36 had less impact on patients with HFS and they may be deleted in future clinical practice. This observation was consistent with previous report by Tan [5,6], who designed a short QoL scale (HFS-7) from subscales of motility (item 4), ADL (items 6, 7), emotional well-being (Item11) and stigma (Items 19, 21, 22). In our study, the majority of HFS-7 items, except items 22, had significant correlation to the metal health (RE, VT, MH and SF) of SF-36 both before and after BTX treatment. This result was similar to Tan's report.

In table 5, half items of HFS-36 with greater mean difference of scores before and after treatment were listed, and the ranking represented the abilities in detecting treatment response to BTX. All the items in subscale of stigma were ranked top, and this result gave us clues that embarrassment and stigma were the major concerns of HFS patients. Except subscales of social support and communication, each subscale contained one or more items that were ranked within top 15. In the previous report of HFS-30 [5], the items were ranked according to *p *value in regression analysis between changes of item scoring and response to BTX treatment. Since only 80 patients enrolled in their study, regression analysis was not adequate to evaluate a scale with 30 items. In addition, the items were ranked by p value rather than R^2 ^value. Therefore, the items selected may be not truly the most sensitive to detect changes of HRQoL and there were some discrepancies compared to our results. Some of their top 10 items were ranked within the last quartile of our ranking, such as item 29, 31 and 33. However, in the short form scale of HFS-7, all the items were among the selected items (table 5), indicating HFS-7 a reliable scale. This result may provide a valuable index to design future short-form scale in different countries. We suggest choosing 10 items from 5 subscales as a modified short scale (HFS 10) for evaluating the HRQoL in Chinese patients with HFS in the future (Table 5).

Compared to SF-36, HFS-36 scale was sensitive and specific to evaluate the mental health in HFS, such as the stigma and embarrassment. Moreover, HFS-36 also detected the impact to physical health, like difficulty in working or reading, which was not observed by SF-36. There were still some limitations in our study. Though HFS-36 is a thorough scale specific for HFS with 8 subscales of HRQoL, items in subscales of social support, cognition and communication were not good enough. In addition, some patients may fell lengthy in answering the questionnaire. It's worth to design a short scale based on table 5 of this study and modified them according to different cultures. The severity of hemifacial spasm fluctuates, only the severity scale may be not enough to detect the treatment response. HFS-36 or a short scale (HFS 10) may be valuable to assess the treatment response and their HRQoL. HFS is common in Asian countries, and validation of a Chinese version of HRQoL scale will be useful in clinical practice among the Chinese populations in Asia.

In conclusion, HFS-36 scale, modified from English version of HFS-30, is the first Chinese version of disease-specific HRQoL scale for HFS. The reliability and validity were good in subscales of motility, ADL, emotion well-being, stigma and bodily discomfort. The HRQoL was significantly improved after BTX assessed by HFS-36 or SF-36. Compared to SF-36, HFS-36 scale was more sensitive and specific to evaluate the HRQoL in HFS.

## Abbreviations

HRQoL: Health-related Quality of Life; HFS: Hemifacial Spasm; ADL: Activities of Daily Living; BTX: Botulinum Toxin; ICC: Intra-Class Correlation; QoL: Quality of Life; PF: Physical Functioning; RP: Role Limitations due to Physical Health; RE: Role Limitations due to Emotional Problems; VT: Vitality; MH: Mental Health; SF: Social Functioning; BP: Bodily Pain; GH: General Health.

## Competing interests

The authors declare that they have no competing interests.

## Authors' contributions

YCH participated in study design and drafted the manuscript. YRW participated in study design and execution. JYF and WCH contributed to statistical analysis. CMC, HSC and RKL were involved in data collection. LSR and STC were responsible for review and critique. All authors read and approved the final manuscript.
